# Model Predictive Controller Approach for Automated Vehicle’s Path Tracking

**DOI:** 10.3390/s23156862

**Published:** 2023-08-01

**Authors:** Ádám Domina, Viktor Tihanyi

**Affiliations:** Department of Automotive Technologies, Budapest University of Technology and Economics, 1111 Budapest, Hungary; tihanyi.viktor@kjk.bme.hu

**Keywords:** automated vehicles, LPV control, model predictive control, steering dynamics, path tracking, vehicle modeling

## Abstract

In this paper, a model predictive control (MPC) approach for controlling automated vehicle steering during path tracking is presented. A (linear parameter-varying) LPV vehicle plant model including steering dynamics is proposed to determine the system evolution matrices. The steering dynamics are modeled in two different ways by using first-order lag and a second-order lag; the application of the first-order system resulted in a slightly more accurate path-following. Additionally, a cascade MPC structure is applied in which two MPCs are used; the second-order steering dynamics are separated from the path-following controller in a second MPC. Both steering system models and the cascade MPC are evaluated in simulation and on a test vehicle. The reference trajectory is calculated based on a fixed predefined path by transforming the necessary path segment to the vehicle ego coordinate system, thereby describing the reference for the path-following task in a novel way. The MPC method computes the optimal steering angle vector at each time step for following the path. The longitudinal dynamics is controlled separately by a PI controller. After simulation evaluation, experimental tests were conducted on a test vehicle on an asphalt surface. Both simulation and experimental results prove the effectiveness of the proposed reference definition method. The effect of the applied steering system models is evaluated. The inclusion of the steering dynamics in the prediction model resulted in a significant increase in controller performance. Finally, the computational requirements of the proposed control and modeling methods are also discussed.

## 1. Introduction

In recent years, collision avoidance systems, advanced driver-assistance systems, and automated vehicle functions have become the most discussed topics in the field of automotive research. The advantages of automated vehicles and their underlying technology include the potential to improve road safety, reduce or eliminate the effect of human errors, reduce pollutant emissions, the number of accidents, and travel times. Automated vehicles are suitable for avoiding collisions by using the steering and braking system conventionally; furthermore, a higher level of vehicle motion control is available for solving an emergency by forcing the vehicle into an unstable state, for example, drifting and controlling the vehicle reliably in this unstable state [1,2].

In the structure of an automated vehicle, the path-following and path-planning modules—which are closely related to each other—are essential to realizing the tasks listed above. In this paper, a model predictive controller (MPC)-based path-following controller structure is proposed to realize path-following scenarios near the handling limit.

The previous research of the authors includes the aim to increase the speed during path-following tasks. There are numerous path-following control solutions as summarized in [3,4,5,6,7,8]. During the research and experimental testing, the authors have concluded that the two most important features required for increasing the speed of the vehicle during path-following are the knowledge of the path ahead of the vehicle and the inclusion of vehicle dynamics into the control law. These attributes are essential to achieve accurate path tracking at higher speeds. The MPC controller makes the realization of both required features possible. Moreover, the MPC can handle constraints; thus, the authors chose MPC for path-following tasks. Furthermore, a third feature—the consideration of the steering dynamics—was identified as an additional requirement for path tracking which, if possible, is needed to be considered in the control law. The MPC makes the incorporation of this additional feature possible as well by choosing the right vehicle model for state-prediction purposes. The adaptive *H_∞_* controller in [6], the nonlinear sliding mode controller in [7], and the adaptive MPC in [8] consider the lateral and orientation error at the center of gravity of the vehicle, but there is no further information about the path in front of the vehicle considered in the control law, while our reference definition method presented below uses information about a greater path segment during the calculation of control input. Furthermore, integrating the steering dynamics into the prediction model is also an added value over [6,7,8].

In the field of path-tracking research, three main types of MPC methods have been implemented and tested which are the linear parameter-varying (LPV-MPC) [9,10], the linear time-varying (LTV-MPC) [11,12], and the nonlinear (NMPC) [11,13] solutions. In the LPV-MPC method, a linear plant model is applied for state prediction. The model does not change during the operation. However, the values can be recalculated at each time step based on the change in a parameter—for example, the velocity—of the vehicle. The change in the parameters is determined by real-time measurements or state estimation techniques. This method has a low computational cos; however, it is not able to handle the nonlinear behavior of the system. In the LTV-MPC method, the plant model is recalculated at each time step based on the online linearization of the applied nonlinear vehicle model. The linearization is always conducted in the current state of the system. Additional transformation of the system matrices and control input command are required. The parameters of the vehicle can be changed in the model as well. The current state which is the basis of the linearization either can be measured or estimated. In the case of nonlinear MPC, the evolution matrices are calculated based on a nonlinear plant model without linearization. This is the most accurate method, yet it is computationally expensive which can limit its applicability, as discussed in [11].

The vehicle model used for state prediction in the MPC controller needs to be chosen according to the control purpose [14,15]. The model should be able to predict future values of the controlled states for which states the desired values are defined by the reference values. Different types of vehicle models are applied in the path-following MPC controllers. In [11], two different types of MPC controllers are used for the path-tracking task: a nonlinear MPC with a nonlinear solver and a LTV-MPC using successive linearization and quadratic cost function. In both cases, the reference values are the heading angle, the yaw-rate and the lateral distance in the global frame, while the control input is the front steering angle. The difference between the two NMPC controllers used in [16] is the level of complexity of the vehicle models applied for state prediction. In one case, the vehicle model is a four-wheel 10 DOF model, while in the other case, it is a simplified 6 DOF bicycle model. The path-following task is conducted by combined steering and braking; hence the control inputs are the front steering angle and the braking torques for each wheel, and the reference is defined for the following states: longitudinal velocity, heading angle, lateral distance in the global frame, and yaw-rate. In [17], a LTV-MPC is applied, a 6 DOF four-wheel vehicle model is successively linearized, the control input is the front steering angle, and the reference states are the yaw-rate and the lateral distance in the global frame. In [18], a LTV-MPC is applied for the path-following task, the control input is the steering angle, and the reference states are the longitudinal and lateral distance in the global frame, and the heading angle. In [19], different controllers for the path following of over-actuated autonomous electric vehicles are presented, where the reference states are the yaw-rate, the sideslip angle, the heading angle, the lateral distance in the global frame, and the longitudinal velocity. The control inputs are the front steering angle and the driving or braking forces for each wheel. In [20], a LTV-MPC is applied in an integrated longitudinal and lateral control scheme where the reference states are the heading angle, the yaw-rate and the lateral distance in the global frame, while the control input is the front steering angle.

In [11,16,17,18,19,20,21], the lateral distance as a reference state is defined in the global frame; hence, the application of the nonlinear vehicle model or successive linearization of the nonlinear model is necessary for state prediction. In this paper, the authors intend to use a linear prediction model without successive linearization; thus, the lateral distance reference is defined in the vehicle’s own frame by transforming the corresponding section of the path using the vehicle’s spatial position and orientation related to the path. With this type of reference definition, and using a linear vehicle model, the nonlinear terms of the vehicle model can be easily omitted from the controller development. Furthermore, the orientation error is defined in a forward-looking way, by transforming the orientation errors to the vehicle’s ego coordinate system, which is a novel reference definition method and is one of the contributions of this paper.

There is a considerable effect that is mostly neglected during the development of path-following controllers [11,16,17,18,19,20,21] which is the consideration of the actuator dynamics—in this case, the dynamics of the steering system. During the previous research of the authors [22], the inclusion of the steering dynamics in the control law is able to significantly improve the performance of the path-following controller. The actuator dynamics can be modeled by a first-order lag [23]. In [9], the dynamics of the steering system are considered by a first-order lag; however, they are not integrated into the prediction model, applied solely in the vehicle model which is used for testing the controller in a simulation environment. In [12], the dynamics of the steering system are modeled by a first-order lag in the nonlinear prediction model which is linearized at each time step in a LTV-MPC structure. Also, a first-order lag is used as a steering system model in [24]. In [25], the steering actuator dynamics are modeled in different ways in order to achieve a smoother path-tracking performance. Furthermore, the backlash is also a considerable concern during the modeling of the steering systems, which can have a significant impact on the performance of a path-following controller. In the applied experimental vehicle, the backlash did not lead to a problem during the tests; hence, its impact is neglected in this article.

### Contribution and Structure of the Paper

In this paper, the authors apply the LPV-MPC method to the path-tracking problem. The stability of the proposed control method is discussed in [26,27]. During the research, the authors intended to apply the proposed LPV-MPC at as high sideslip angles as possible. As expected by the authors, the proposed controller is applicable until the sideslip angles of the tires are in the linear region of the tire characteristics because a linearized vehicle model coupled with a linear tire model is used for state prediction. The phenomenon was confirmed during the experimental tests. The desired path-tracking accuracy can be reached by defining the path-following task optimally, in a novel way, and with the consideration of the steering dynamics in the state prediction. The plant model is calculated at each time step based on the measured states. As all of the states can be measured on the test vehicle, there is no need for using a state estimator. While in articles [9,10,11,12,13,28] the applied methods are LTV-MPC or NLMPC coupled with constraints on the sideslip angles, in this paper, the sideslip angle is not constrained, yet similarly, a high performance is reached by the optimal description of the path-tracking problem and the inclusion of the steering dynamics in the plant model used for state prediction.

In this article, a novel reference definition method is presented which determines both the reference lateral displacement and the reference angular orientation of the vehicle for a finite horizon in front of the vehicle. In the existing works [9,29], the reference trajectory is not defined in a forward-looking way, although if so, then solely the lateral displacement reference is given by different forward-looking methods [10,17].

The path-following performance is aimed to be enhanced by considering the steering system dynamics in the plant model. In this paper, the steering system is modeled both as a first-order and as a second-order system. The steering system models are identified on the test vehicle. In [30], a cascade MPC structure is proposed to reduce the computational requirements of the path-following controller and for a more accurate tracking realization. In this paper, additionally, the proposed cascade MPC structure is also examined; thereby, four different controllers are evaluated: a controller excluding the steering dynamics, a controller using first-order steering system dynamics, another using second-order dynamics, and a fourth one realizing the cascade MPC using second-order steering dynamics, and in which the controller of the steering dynamics is separated from the path-following controller. The performance of the controllers and the effect of the steering models are evaluated and compared in a simulation environment and on a test vehicle.

In summary, the contribution of this paper is twofold: a novel reference definition method is given in a forward-looking way, and, furthermore, the effect of the integration of different steering system models into the plant model of a MPC, and a cascade MPC structure on the path-following are evaluated in both simulation and experimental environments.

The structure of this paper is as follows. Section 2 presents the vehicle model used during the in-simulation development of the controller, and the different types of vehicle models applied in the MPC for state prediction, including the steering dynamics. Furthermore, Section 2 briefly presents the experiment-based identification of the parameters used in the nonlinear tire model. Section 3 describes the reference path-generation method. Section 4 and Section 5 explain the MPC problem formulation including the derivation of the cost function which needs to be minimized by the MPC by solving the quadratic programming (QP) problem, and the cascade MPC formulation, respectively. In Section 6, the simulation and experimental results are examined in detail and a profound comparison is made. Finally, Section 7 summarizes the concluding remarks and further plans.

## 2. Vehicle Modeling

In this section, the test vehicle setup, the applied vehicle models, and the parameter identification are presented. In this paper, the authors use two different types of vehicle models. One of the models is a four-wheel vehicle model in which nonlinear tire models are used at each wheel. This model was used during the testing and validation process of the MPC in a simulation environment. The other model is a linearized dynamic bicycle model which is applied to the MPC plant model for state prediction.

### 2.1. Test Vehicle Setup

The used vehicle platform is a BMW M2 Competition series production coupe car on which the necessary modifications were made to prepare it for executing the measurements. The vehicle is powered by a twin-turbocharged 3.0 L straight-six engine which produces 302 kW of performance and 550 Nm of torque. It has rear-wheel drive and a 7-speed dual-clutch automatic transmission. The vehicle was prepared for the tests by equipping it with a steering robot and throttle-by-wire systems.

**Figure 1:**
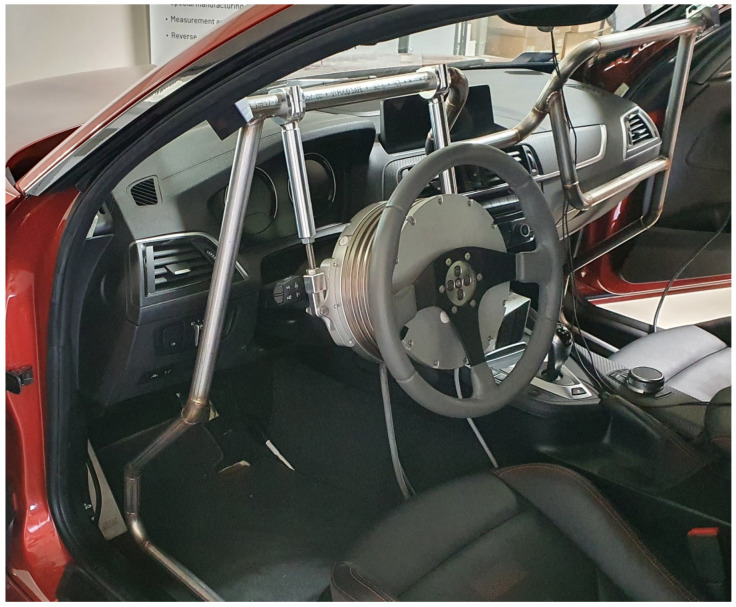
Steering robot installed in the test vehicle.

For the MPC implementation, online data acquisition, and for the control of the actuators, a dSpace MicroAutoBox II 1401 was used. The MPC controller was built in the MATLAB Simulink environment from which C code was generated and uploaded to the MicroAutoBox. As the maximum time requirement of the MPC is less than 0.35 ms, the entire control model runs with a 1 ms time step. The steer-by-wire function is realized by a FER-201 steering robot used in angle control mode, as per the installation shown in Figure 1. The steering angle requested by the MPC is realized by the separated PID controller of the steering robot subsystem. All of the sensors and actuators are connected to the MicroAutoBox via the CAN interface.

The accelerator pedal of the vehicle is removed and its signal is emulated by a digital–analog converter which is built into the MicroAutoBox. The velocity of the vehicle is controlled separately from the lateral motion control by a PI controller. Hence, the transmission is used in automatic mode, the velocity demand is sent to the PI controller, the controlled variable is assigned to the accelerator pedal position, and the feedback variable is assigned to the velocity of the vehicle measured via its CAN bus.

For self-localization, an iMAR dual-antenna GNSS system with a built-in inertial measurement unit (IMU) and with RTK correction was used. All of the required states were measured or calculated from the signals provided by the GNSS system. The signals consist of the X and Y coordinates in UTM coordinate system, the heading angle, the yaw-rate, and the sideslip angle of the vehicle. Furthermore, all of the signal values are provided at the center of gravity (C.G.) of the vehicle, and the sideslip angles of the tires are calculated from the vehicle sideslip angle β provided by the GNSS system. Additional signals are read from the CAN bus of the vehicle and converted to FMS-Standard messages by an FMS Gateway. The CAN signals used during both measurement and system identification are the steering angle, the yaw-rate, the engine torque, the vehicle velocity, and the accelerator pedal position.

### 2.2. Vehicle Model for Simulation

**Figure 2:**
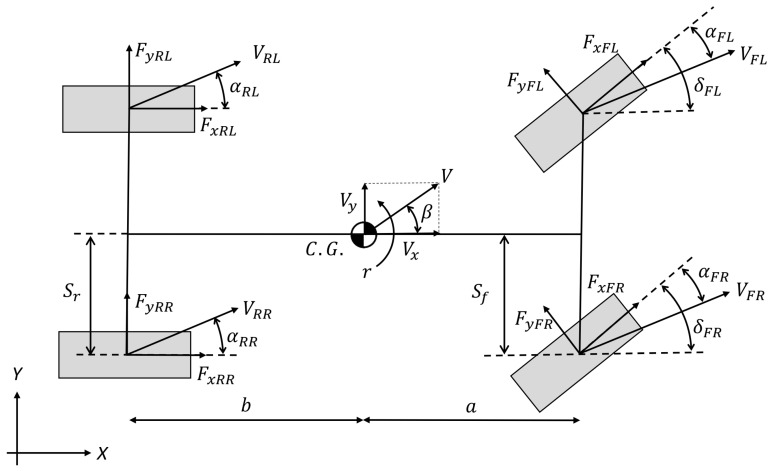
Four-wheel vehicle model with applied symbols.

For testing the controller in a simulation environment, a four-wheel dynamic vehicle model was used. The vehicle model only considers the planar dynamics of the vehicle, while the rolling and pitching dynamics are neglected as they are not relevant to the current problem. Equations (1)–(6), describe the longitudinal, lateral and yaw movement of the vehicle, based on Newton’s second law:

(1) $$a_{x}=\frac{1}{m}F_{x}^{V}+V_{y}r$$

(2) $$a_{y}=\frac{1}{m}F_{y}^{V}-V_{x}r$$

(3) $$\dot{r}=\frac{1}{I_{z}}M_{z}$$

(4) $$F_{x}^{V}=F_{xRL}+F_{xRR}+F_{yFL}\sin\left(\delta_{FL}\right)+F_{yFR}\sin\left(\delta_{FR}\right)+F_{xFL}\cos\left(\delta_{FL}\right)+F_{xFR}\cos\left(\delta_{FR}\right)$$

(5) $$F_{y}^{V}=F_{yRL}+F_{yRR}+F_{xFL}\sin\left(\delta_{FL}\right)+F_{xFR}\sin\left(\delta_{FR}\right)+F_{yFL}\cos\left(\delta_{FL}\right)+F_{yFR}\cos\left(\delta_{FR}\right)$$

(6) $$M_{z}=-b(F_{yRL}+F_{yRR})+s_{f}(-F_{xFL}\sin\left(\delta_{FL}\right)+F_{xFR}\sin\left(\delta_{FR}\right))+s_{r}\left(-F_{xRL}+F_{xRR}\right)+a\left(F_{yFL}\cos\left(\delta_{FL}\right)\;+F_{yFR}\cos\left(\delta_{FR}\right)\right)$$

where *a_x_* is the longitudinal acceleration, *a_y_* is the lateral acceleration, *F_yFL_*, *F_yFR_*, *F_yRL_* and *F_yRR_* are the lateral tire forces at the front and the rear wheels, at the left and the right side, respectively, *F_xFL_*, *F_xFR_*, *F_xRL_* and *F_xRR_* are the longitudinal tire forces at the front and the rear wheels, at the left and the right side, respectively, where *F_xRL_* and *F_xRR_* are considered equal, *m* is the mass of the vehicle, *r* is the yaw-rate, *a* and *b* are the distances from the front and the rear wheel to the center of gravity, respectively. Furthermore, *s_f_* and *s_r_* are the front and the rear track, respectively, *I_z_* is the moment of inertia around axis *z*, *V_x_* is the longitudinal velocity, *V_y_* is the lateral velocity in the ego coordinate system, *δ_FL_* and *δ_FR_* are the front steering angle at the left and at the right side, respectively, and finally α*_FL_*, *α_FR_*, *α_RL_* and *α_RR_* are the front and rear sideslip angles at the left and the right sides, respectively, as shown in Figure 2. The values of *δ_FL_* and *δ_FR_* are different, the ratio of the steering system is identified via measurements conducted with the test vehicle. The rear wheels are assumed to be unable to be steered.

**Figure 3:**
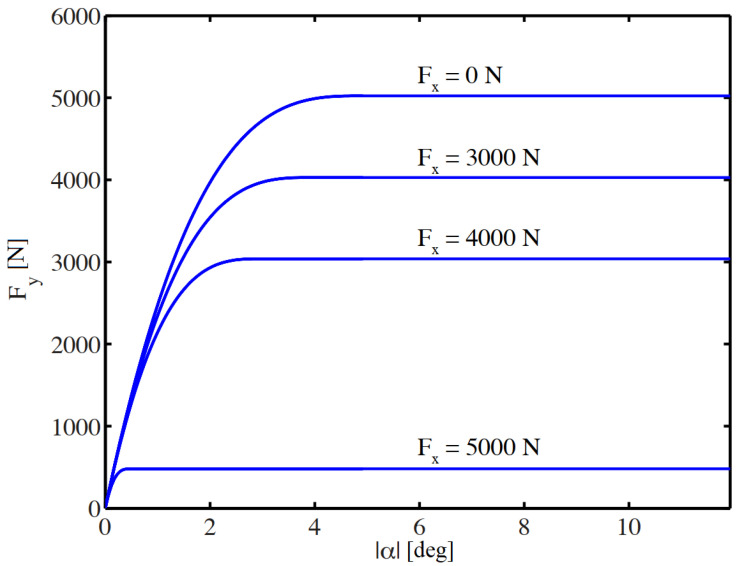
Lateral force characteristics of the brush tire model as a function of longitudinal force and sideslip angle.

The brush tire model [31] is used to calculate the lateral tire forces by Equations (7)–(9), where *C_α_* is the tire cornering stiffness, *α* is the tire sideslip angle, *µ* is the friction coefficient and *F_z_* is the vertical tire load. The model includes a *ζ* derating factor as suggested in [32] to consider the coupling between lateral and longitudinal forces. The calculation of the forces is based on the friction circle. The longitudinal traction force acts at the rear wheels hence the derating factor is only applied to the rear wheels. The variable *α_sl_* indicates the boundary of the sideslip angle where the entire tire is fully saturated, in which state the further increase in the sideslip angle will not increase the lateral force. At the front wheels, zero longitudinal force is assumed. In this case, the lateral force can be calculated by Equations (7) and (8), substituting *ζ =* 1 into the equations (Figure 3).

(7) $$F_{y}=\left\{\begin{array}{c} -C_{\alpha }tan\left(\alpha \right)+\frac{C_{\alpha }^{2}}{3\zeta \mu F_{z}}\left|tan\left(\alpha \right)\right|tan\left(\alpha \right)-\frac{C_{\alpha }^{3}}{27\zeta^{2}\mu^{2}F_{z}^{2}}tan^{3}\left(\alpha \right),\;\;\left|\alpha \right|\leq \alpha_{sl}\\ -\zeta \mu F_{z}sgn\left(\alpha \right),\;\;\left|\alpha \right|>\alpha_{sl} \end{array}\right.$$

(8) $$\alpha_{sl}=\frac{3\zeta \mu F_{z}}{C_{\alpha }}$$

(9) $$\zeta =\frac{\sqrt{{\left(\mu_{R}F_{zR}\right)}^{2}-F_{x}^{2}}}{\mu_{R}F_{zR}}$$

The sideslip angles of the individual wheels are calculated by Equations (11) and (12) at the front and rear wheels, respectively.

(10a) $$v_{i}=\left[\begin{array}{c} v_{xi}\\ v_{yi}\\ v_{zi} \end{array}\right]=\left[\begin{array}{c} V_{x}\\ V_{y}\\ V_{z} \end{array}\right]+\left[\begin{array}{c} 0\\ 0\\ r \end{array}\right]\times P_{i}$$

(10b) $$P_{FL}={\left[\begin{array}{ccc} a & s_{f} & 0 \end{array}\right]}^{T},\;P_{FR}={\left[\begin{array}{ccc} a & -s_{f} & 0 \end{array}\right]}^{T}$$

(10c) $$P_{RL}={\left[\begin{array}{ccc} -b & s_{r} & 0 \end{array}\right]}^{T},\;P_{RR}={\left[\begin{array}{ccc} -b & -s_{r} & 0 \end{array}\right]}^{T}$$

(11) $$\alpha_{FL}=arctan\frac{v_{yFL}}{v_{xFL}}-\delta_{FL},\;\alpha_{FR}=arctan\frac{v_{yFR}}{v_{xFR}}-\delta_{FR}$$

(12) $$\alpha_{RL}=arctan\frac{v_{yRL}}{v_{xRL}},\;\alpha_{RR}=arctan\frac{v_{yRR}}{v_{xRR}}$$

where *v_i_* is the velocity vector (*i = FL ˅ FR ˅ RL ˅ RR*) of the wheels, calculated by using the [*V_x_,V_y_,V_z_*]*^T^* velocity vector of the vehicle at the C.G., the yaw-rate, and the *P_i_* location vector of the wheels.

**Figure 4:**
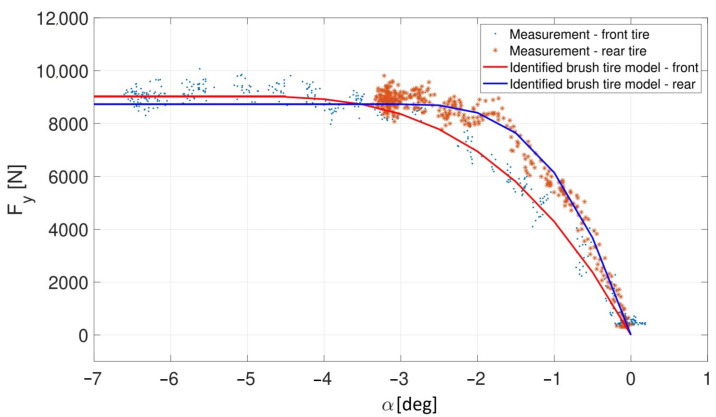
Front and rear tire characteristics [33].

The vehicle parameters *m* and *I_z_* are measured, while the cornering stiffness of the tires is identified by ramp steer tests in [33]. The tire characteristics fitted to the respective test results are shown in Figure 4.

### 2.3. Vehicle Models Applied by the MPC

**Figure 5:**
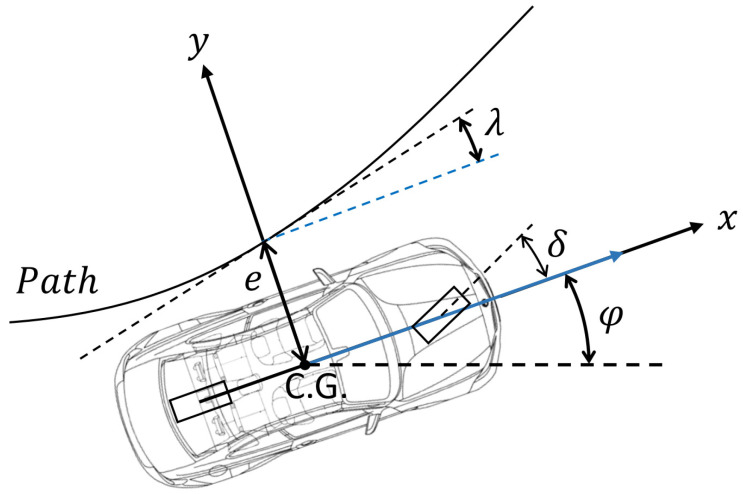
Lateral and orientation error.

The interpretation of the lateral and orientation error is shown in Figure 5. The lateral error *e* is the distance measured between the center of gravity of the vehicle and the closest point to it on the path.

The orientation error *λ* calculated at the same path point as the distance error is the difference between the current heading angle *φ* of the vehicle and the tangent of the path at the closest point to the center of gravity.

In this paper, the authors apply a linearized bicycle model for state prediction. The model (Equation (13)) describes the lateral dynamics of the vehicle where the states are the lateral error, the derivative of the lateral error, the orientation error and the derivative of the orientation error [34].

(13) $$\dot{x}=Ax+Bu$$

$$A=\left[\begin{array}{cccc} 0 & 1 & 0 & 0\\ 0 & -\frac{2C_{\alpha f}+2C_{\alpha r}}{mV_{x}} & \frac{2C_{\alpha f}+2C_{\alpha r}}{m} & \frac{-2C_{\alpha f}l_{f}+2C_{\alpha r}l_{r}}{mV_{x}}\\ 0 & 0 & 0 & 1\\ 0 & -\frac{2C_{\alpha f}l_{f}-2C_{\alpha r}l_{r}}{I_{z}V_{x}} & \frac{2C_{\alpha f}l_{f}-2C_{\alpha r}l_{r}}{I_{z}} & -\frac{2C_{\alpha f}l_{f}^{2}-2C_{\alpha r}l_{r}^{2}}{I_{z}V_{x}} \end{array}\right]$$

$$B=\left[\begin{array}{c} \begin{array}{c} 0\\ \frac{2C_{\alpha f}}{m} \end{array}\\ \begin{array}{c} 0\\ \frac{2C_{\alpha f}l_{f}}{I_{z}} \end{array} \end{array}\right]$$

The state vector is *x =* [$e_{1}\;{\dot{e}}_{1}\;e_{2}\;{\dot{e}}_{2}$]*^T^*, where *e_1_* is the lateral error, *e_2_* is the orientation error, and the control input *u* is the steering angle. Based on Equation (13), the future states of the vehicle can be predicted. As shown in Figure 4, the linear characteristic of the tires are between 0 and 3.5 degrees of the sideslip angle at the front wheels, and 0 and 2 degrees of the sideslip angle at the rear wheels; hence, the prediction is acceptable until the sideslip angles are in these ranges, respectively. Above these values, the prediction becomes incorrect due to the neglect of the tire nonlinearities. Accordingly, the controller is expected to be applicable approximately up to a 3.5 degrees/2 degrees of front/rear sideslip angle, whose expectation is proved by the experimental results presented in Section 6.

In this article, the dynamics of the steering system are modeled in two different ways: by a first-order lag (1TP) Equation (14) or by a second-order lag (2TP) Equation (16), for which both models can be easily included in the prediction model.

(14) $${\dot{\delta }}_{act}=-\frac{1}{T_{st}}\delta_{act}+\frac{1}{T_{st}}\delta_{ref}$$

**Figure 6:**
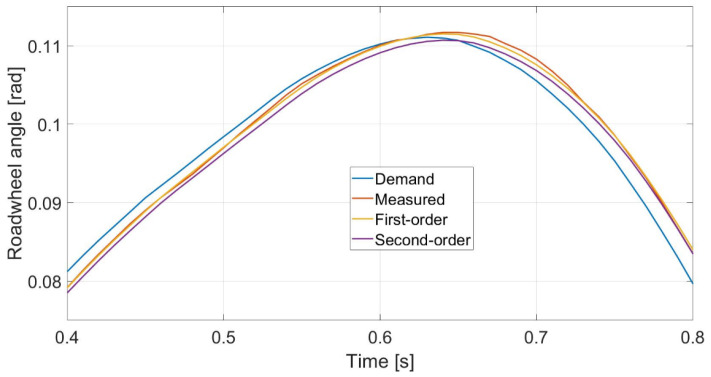
Reference tracking of the steering robot, the identified first-order and second-order systems.

The first-order steering dynamics Equation (14) can be written as a transfer function (Equation (15)).

(15) $$\delta_{act}=\frac{1}{T_{st}\cdot s+1}\delta_{ref}$$

(16) $$\delta_{act}=\frac{b}{s^{2}+s\cdot a_{st1}+a_{st0}}\delta_{ref}$$

where $\delta_{act}$ is the actual steering angle, $\delta_{ref}$ is the reference steering angle demanded by the controller—both are road wheel angle—and $T_{st}$ is the time constant of the first-order steering system, while *a_st_*_1_, *a_st_*_0_ and *b* are the parameters of the second-order steering system. The *T_st_* time constant and the *a_st_*_1_, *a_st_*_0_, *b* parameters are identified based on measurement results. The identified steering system models are compared with the measured steering system; the result is shown in Figure 6. Because the measured and the identified systems show a high degree of coincidence, Figure 6 only shows the results in a small time window in order to achieve better visibility. As shown in Figure 6, the lag between signals ‘Measured’ and ‘Demand’ is small because of the high torque of the steering robot.

In the case of using the first-order steering dynamics, the dynamic model of vehicle Equation (17) is identical with Equation (13); however, the state vector is supplemented with a fifth state, the actual steering angle *δ_act_*, yields $x={\left[e_{1}\;{\dot{e}}_{1}\;e_{2}\;{\dot{e}}_{2}\;\delta_{act}\right]}^{T}$.

(17) $$\dot{x}=Ax+Bu$$

$$A=\left[\begin{array}{ccccc} 0 & 1 & 0 & 0 & 0\\ 0 & -\frac{2C_{\alpha f}+2C_{\alpha r}}{mV_{x}} & \frac{2C_{\alpha f}+2C_{\alpha r}}{m} & \frac{-2C_{\alpha f}l_{f}+2C_{\alpha r}l_{r}}{mV_{x}} & \frac{2C_{\alpha f}}{m}\\ 0 & 0 & 0 & 1 & 0\\ 0 & -\frac{2C_{\alpha f}l_{f}-2C_{\alpha r}l_{r}}{I_{z}V_{x}} & \frac{2C_{\alpha f}l_{f}-2C_{\alpha r}l_{r}}{I_{z}} & -\frac{2C_{\alpha f}l_{f}^{2}-2C_{\alpha r}l_{r}^{2}}{I_{z}V_{x}} & \frac{2C_{\alpha f}l_{f}}{I_{z}}\\ 0 & 0 & 0 & 0 & -\frac{1}{T_{st}} \end{array}\right]$$

$$B=\left[\begin{array}{c} \begin{array}{c} 0\\ 0 \end{array}\\ \begin{array}{c} 0\\ \begin{array}{c} 0\\ \frac{1}{T_{st}} \end{array} \end{array} \end{array}\right]$$

As seen in Equations (13) and (17), the variable in the vehicle model is the longitudinal velocity *V_x_*. The prediction model is updated in every time step using the current *V_x_*.

In the case of using the second-order steering dynamics, the state-space representation of the vehicle in Equation (13) is expanded from four to six states by incorporating the actual steering angle and the angular velocity of the actual steering angle ${\dot{\delta }}_{act}$ into the state vector: *x =* [$e_{1}\;{\dot{e}}_{1}\;e_{2}\;{\dot{e}}_{2}$ $\delta_{act}\;{\dot{\delta }}_{act}$]*^T^*. Finally, the vehicle model containing the second-order steering dynamics is defined in (18):

(18) $$\dot{x}=Ax+Bu$$

$$A=\left[\begin{array}{cccccc} 0 & 1 & 0 & 0 & 0 & 0\\ 0 & -\frac{2C_{\alpha f}+2C_{\alpha r}}{mV_{x}} & \frac{2C_{\alpha f}+2C_{\alpha r}}{m} & \frac{-2C_{\alpha f}l_{f}+2C_{\alpha r}l_{r}}{mV_{x}} & \frac{2C_{\alpha f}}{m} & 0\\ 0 & 0 & 0 & 1 & 0 & 0\\ 0 & -\frac{2C_{\alpha f}l_{f}-2C_{\alpha r}l_{r}}{I_{z}V_{x}} & \frac{2C_{\alpha f}l_{f}-2C_{\alpha r}l_{r}}{I_{z}} & -\frac{2C_{\alpha f}l_{f}^{2}-2C_{\alpha r}l_{r}^{2}}{I_{z}V_{x}} & \frac{2C_{\alpha f}l_{f}}{I_{z}} & 0\\ 0 & 0 & 0 & 0 & 0 & 1\\ 0 & 0 & 0 & 0 & -a_{st0} & -a_{st1} \end{array}\right]$$

$$B=\left[\begin{array}{c} \begin{array}{c} 0\\ 0 \end{array}\\ \begin{array}{c} 0\\ \begin{array}{c} 0\\ \begin{array}{c} 0\\ b \end{array} \end{array} \end{array} \end{array}\right]$$

The experimental tests are conducted by using both vehicle models; the comparison of the results shows that those models which include steering dynamics are significantly more accurate and stable.

## 3. Reference Generation

As explained in Section 2, the authors intend to apply a linear prediction model and avoid the application of nonlinear prediction models or successive linearization techniques. Hence, the reference path which needs to be followed is defined by the corresponding states of the prediction model; thus, the reference lateral error *e*_1_ and the orientation error *e*_2_ values are given in a novel forward-looking way. As a result of defining the reference in this way, the path is defined by the desired lateral position values of the path points and by the orientation angle values. The distances of the lateral position values of the transformed reference path and the angles are considered as an error viewed from the current state of the vehicle in the vehicle’s ego coordinate system. The lateral error and the orientation error are the references for the first and the third elements of the state vector *x*, respectively.

(19a) $$\kappa =\lambda_{1}+\varphi$$

(19b) $$H=\left[\begin{array}{c} 0\\ e_{1} \end{array}\right]$$

(19c) $$P_{ref,i}=H+\left[\begin{array}{cc} cos\left(\kappa \right) & -sin\left(\kappa \right)\\ sin\left(\kappa \right) & cos\left(\kappa \right) \end{array}\right]P_{i}$$

To calculate these error values, the path is transformed from the global coordinate system into the ego coordinate system Equation (19) where *κ* is the rotation angle, *λ_1_* is the orientation error at the closest point to the center of gravity, *H* is the offset vector, *e_1_* is the lateral error at the closest point to the center of gravity, *P_ref,i_* is the transformed point and *P_i_* is the original path point, and *i =* 2*… N_p_*.

**Figure 7:**
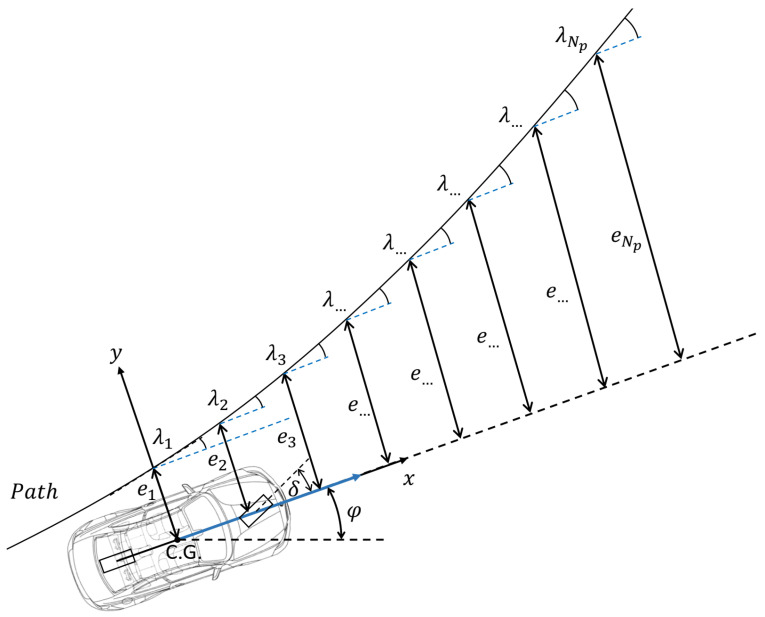
Reference lateral error and orientation error in the ego frame.

Figure 7 shows the principle of the reference definition where *(X,Y)* is the global frame, *(x,y)* is the ego frame, while *e*_1_*, e*_2_*, … e_Np_* are the lateral error references, and *λ*_1_*, λ*_2_*, …. λ_Np_* are the orientation error references. The orientation error references are calculated at the same points as the lateral error references. The controller uses *N_p_* path points as references for the calculation of the optimal sequence of the steering demand where *N_p_* is the prediction horizon of the controller. The desired lateral error and orientation error are calculated at each *N_p_* path point. Finally, the reference for each point is a *ϕ*-vector equation (see Equation (20a)) where *e_i_* is the reference lateral error for the *i_th_* path point and *λ_i_* is the reference orientation error for the *i_th_* path point. The reference vector for the entire prediction horizon is a stacked matrix *Φ* (Equation (20b)) with a dimension of 2 *× N_p_*.

Defining the reference path in this way, the desired *(X*,*Y)* values are transformed into the desired errors which are coherent with the states of the applied prediction model. The presented reference definition method is considered as one of the contributions of this paper, especially the forward-looking definition of angular errors.

(20a) $$\phi_{i}=\left[\begin{array}{c} e_{i}\\ \lambda_{i} \end{array}\right]$$

(20b) $$\Phi ={\left[\begin{array}{cccc} \phi_{1}^{T} & \phi_{2}^{T} & \cdots & \phi_{N_{p}}^{T} \end{array}\right]}^{T}=\left[\begin{array}{ccccccc} e_{1} & \lambda_{1} & e_{2} & \lambda_{2} & \cdots & e_{N_{p}} & \lambda_{N}{}_{p} \end{array}\right]{\;}^{T}$$

## 4. MPC Formulation

In the applied control structure, the vehicle model and the reference matrix are updated in every time step based on the current state of the vehicle regarding the path. Afterward, the evolution matrices, the reference matrix, and the current vehicle state are coupled in the QP cost function from which the optimal steering demand vector u is calculated. The QP problem is solved by the built-in MATLAB solver.

The evolution matrices Equation (21) of the system are calculated by the widely used method [35] where *A* is the state matrix, *B* is the control input matrix, *u* is the control input—in this case, the steering angle—and *x* is the vector of the state variables.

(21) $$\overset{\hat{\bar{x}}}{\overbrace{\left[\begin{array}{c} x\left(k+1\right)\\ x\left(k+2\right)\\ \vdots \\ x\left(k+N_{p}\right) \end{array}\right]}}=\overset{\overset{=}{A}}{\overbrace{\left[\begin{array}{c} A\\ A^{2}\\ \vdots \\ A^{N_{p}} \end{array}\right]}}x\left(k\right)+\overset{\overset{=}{B}}{\overbrace{\left[\begin{array}{ccc} \begin{array}{cc} B & 0 \end{array} & \cdots & 0\\ \begin{array}{cc} AB & B \end{array} & \cdots & 0\\ \begin{array}{cc} \vdots & \vdots \end{array} & \ddots & \vdots \\ \begin{array}{cc} A^{N_{p}-1}B & A^{N_{p}-2}B \end{array} & \cdots & B \end{array}\right]}}\overset{\bar{u}}{\overbrace{\left[\begin{array}{c} u\left(k\right)\\ u\left(k+1\right)\\ \vdots \\ u\left(k+N_{p}-1\right) \end{array}\right]}}$$

Both the deviation of the vehicle states from the reference states and the magnitude of the applied control input need to be penalized by the cost function. Thus, an error vector *z* is created for penalizing the deviation of the states (Equation (22)) where *z*_1_ and *z*_2_ are the error terms for the deviation of the lateral error and the orientation error, respectively.

(22) $$z\left(k\right)=\left[\begin{array}{c} z_{1}\left(k\right)\\ z_{2}\left(k\right) \end{array}\right]=\left[\begin{array}{c} e_{1,ref}\left(k\right)-e_{1}\left(k\right)\\ e_{2,ref}\left(k\right)-e_{2}\left(k\right) \end{array}\right]=Cx+D\phi =\;\left[\begin{array}{cccc} -1 & 0 & 0 & 0\\ 0 & 0 & -1 & 0 \end{array}\right]\left[\begin{array}{c} e_{1}\left(k\right)\\ {\dot{e}}_{1}\left(k\right)\\ e_{2}\left(k\right)\\ {\dot{e}}_{2}\left(k\right) \end{array}\right]+\left[\begin{array}{cc} 1 & 0\\ 0 & 1 \end{array}\right]\left[\begin{array}{c} e_{1,ref}\left(k\right)\\ e_{2,ref}\left(k\right) \end{array}\right]$$

In (22), *C* is the output matrix, *D* is the selection matrix for the reference, *C* and *D* are constructed in correspondence with the state vector *x* ∈ *R*^4^, and the reference vector *ϕ*, respectively, while *e*_1*,ref*_ *(k)* is the reference lateral error and *e*_2*,ref*_ *(k)* is the reference orientation error. In that case, when the enhanced model including the steering dynamics is used, matrix *C* is changed according to Equation (23). Based on which steering system model is used, the enhanced state vector is *x* ∈ *R*^5^ for the first-order steering model (Equation (23a)), and is *x* ∈ *R*^6^ for the second-order steering model (Equation (23b)).

(23a) $$C_{firs-order}=\left[\begin{array}{ccccc} -1 & 0 & 0 & 0 & 0\\ 0 & 0 & -1 & 0 & 0 \end{array}\right]$$

(23b) $$C_{second-order}=\left[\begin{array}{ccccc} -1 & 0 & 0 & 0 & \begin{array}{cc} 0 & 0 \end{array}\\ 0 & 0 & -1 & 0 & \begin{array}{cc} 0 & 0 \end{array} \end{array}\right]$$

The error vector *z* is extended for the entire prediction horizon Equation (24),

(24) $$\overset{\hat{\bar{z}}}{\overbrace{\left[\begin{array}{c} z\left(k+1\right)\\ z\left(k+2\right)\\ \vdots \\ z\left(k+N_{p}\right) \end{array}\right]}\;}\;=+\overset{\overset{=}{C}\;}{\overbrace{\left[\begin{array}{ccc} \begin{array}{cc} C & 0\\ 0 & C \end{array} & \cdots & \begin{array}{c} 0\\ 0 \end{array}\\ \vdots & \ddots & \vdots \\ \begin{array}{cc} 0 & 0 \end{array} & \cdots & C \end{array}\right]}\;}\;\overset{\hat{\bar{x}}}{\overbrace{\left[\begin{array}{c} x\left(k\right)\\ x\left(k+1\right)\\ \vdots \\ x\left(k+N_{p}\right) \end{array}\right]}\;}\;\;\;+\;\overset{\overset{=}{D}\;}{\overbrace{\left[\begin{array}{ccc} \begin{array}{cc} D & 0\\ 0 & D \end{array} & \cdots & \begin{array}{c} 0\\ 0 \end{array}\\ \vdots & \ddots & \vdots \\ \begin{array}{cc} 0 & 0 \end{array} & \cdots & D \end{array}\right]}\;}\;\overset{\hat{\bar{\Phi }}}{\overbrace{\left[\begin{array}{c} \phi \left(k+1\right)\\ \phi \left(k+2\right)\\ \vdots \\ \phi \left(k+N_{p}\right) \end{array}\right]}\;}\;$$

where $\overset{=}{C}\in R^{2N_{p}\times 4N_{p}}$ or $\overset{=}{C}\in R^{2N_{p}\times 5N_{p}}$ or $\overset{=}{C}\in R^{2N_{p}\times 6N_{p}}$—according to the applied steering system model—is the output hypermatrix, and $\overset{=}{D}\in R^{2N_{p}\times 2N_{p}}$ is the selection hypermatrix for the reference. Both $\overset{=}{C}$ and $\overset{=}{D}$ are stacked diagonal matrices derived from the *C* and *D* matrices.

The cost function is formulated by using the state vector and the error matrix for penalizing the state deviation, and the control input matrix for penalizing the magnitude of the controlled variable. The general form of the cost functions for QP problems is defined as Equation (25) where ${\overset{=}{Q}}_{x}\in R^{4N_{p}\times 4N_{p}}$ or ${\overset{=}{Q}}_{x}\in R^{5N_{p}\times 5N_{p}}$ or ${\overset{=}{Q}}_{x}\in R^{6N_{p}\times 6N_{p}}$– according to the applied steering system model—and ${\overset{=}{Q}}_{z}\in R^{2N_{p}\times 2N_{p}}$ are weight matrices for the state deviation, and $\overset{=}{R}\in R^{N_{p}\times N_{p}}$ is the weight matrix for the controlled variable, while ${\hat{\bar{x}}}^{T}\in R^{N_{p}\times 4N_{p}}$ or ${\hat{\bar{x}}}^{T}\in R^{N_{p}\times 5N_{p}}$ or ${\hat{\bar{x}}}^{T}\in R^{N_{p}\times 6N_{p}}$—according to the applied steering system model—and ${\hat{\bar{z}}}^{T}\in R^{N_{p}\times 2N_{p}}$ are the stacked vectors of the predicted states and errors, respectively, and ${\bar{u}}^{T}\in R^{N_{p}\times N_{p}}$ is the optimal control input vector which is the solution of the QP problem.

(25) $$J\left(k\right)=\frac{1}{2}\left({\hat{\bar{x}}}^{T}{\overset{=}{Q}}_{x}\hat{\bar{x}}+{\hat{\bar{z}}}^{T}{\overset{=}{Q}}_{z}\hat{\bar{z}}+{\bar{u}}^{T}\overset{=}{R}\bar{u}\right)$$

(26a) $${\overset{=}{Q}}_{x}=\left[\begin{array}{cccc} Q_{x} & 0 & \cdots & 0\\ 0 & Q_{x} & \ldots & 0\\ \vdots & \vdots & \ddots & \vdots \\ 0 & 0 & \cdots & Q_{x} \end{array}\right]$$

(26b) $${\overset{=}{Q}}_{z}=\left[\begin{array}{cccc} Q_{z} & 0 & \cdots & 0\\ 0 & Q_{z} & \ldots & 0\\ \vdots & \vdots & \ddots & \vdots \\ 0 & 0 & \cdots & Q_{z} \end{array}\right]$$

(26c) $$\overset{=}{R}=\left[\begin{array}{cccc} R & 0 & \cdots & 0\\ 0 & R & \ldots & 0\\ \vdots & \vdots & \ddots & \vdots \\ 0 & 0 & \cdots & R \end{array}\right]$$

where ${\overset{=}{Q}}_{x}$, ${\overset{=}{Q}}_{z}$ and $\overset{=}{R}$ are diagonal matrices Equation (26) created by using matrices *Q_x_*, *Q_z_* and scalar *R* (Equation (19)) where *Q_x_* is changed according to the applied vehicle model, i.e., according to the dimensions of the state vector *x*. Using the model without steering dynamics, *Q_x_ ∈ R*^4×4^ (Equation (20a))—when *x = [*$e_{1}\;{\dot{e}}_{1}\;e_{2}\;{\dot{e}}_{2}$*]^T^*—while using the enhanced model including the first-order steering dynamics, *Q_x_* ∈ *R*^5×5^ (Equation (27b))—when *x = [*$e_{1}\;{\dot{e}}_{1}\;e_{2}\;{\dot{e}}_{2}$ $\delta_{act}$*]^T^*—and when using the second-order steering dynamics, *Q_x_* ∈ *R^6×6^* (Equation (27c))—when *x = [*$e_{1}\;{\dot{e}}_{1}\;e_{2}\;{\dot{e}}_{2}$ $\delta_{act}\;{\dot{\delta }}_{act}$*]^T^*.

(27a) $$Q_{x}=\left[\begin{array}{cccc} q_{x1} & 0 & 0 & 0\\ 0 & 0 & 0 & 0\\ 0 & 0 & q_{x2} & 0\\ 0 & 0 & 0 & 0 \end{array}\right]$$

(27b) $$Q_{x}=\left[\begin{array}{ccccc} q_{x1} & 0 & 0 & 0 & 0\\ 0 & 0 & 0 & 0 & 0\\ 0 & 0 & q_{x2} & 0 & 0\\ 0 & 0 & 0 & 0 & 0\\ 0 & 0 & 0 & 0 & 0 \end{array}\right]$$

(27c) $$Q_{x}=\left[\begin{array}{ccccc} q_{x1} & 0 & 0 & 0 & \begin{array}{cc} 0 & 0 \end{array}\\ 0 & 0 & 0 & 0 & \begin{array}{cc} 0 & 0 \end{array}\\ 0 & 0 & q_{x2} & 0 & \begin{array}{cc} 0 & 0 \end{array}\\ 0 & 0 & 0 & 0 & \begin{array}{cc} 0 & 0 \end{array}\\ \begin{array}{c} 0\\ 0 \end{array} & \begin{array}{c} 0\\ 0 \end{array} & \begin{array}{c} 0\\ 0 \end{array} & \begin{array}{c} 0\\ 0 \end{array} & \begin{array}{cc} 0 & 0\\ 0 & 0 \end{array} \end{array}\right]$$

In Equation (27), *q_x_*_1_ and *q_x_*_2_ are the weights of the lateral error and the orientation error, respectively. *Q_z_* and *R* are identical for both vehicle models Equation (28) where *q_z_*_1_ and *q_z_*_2_ are the weights of the deviation from the reference lateral error and the orientation error, respectively, and *r* is the weight of the control input.

(28a) $$Q_{z}=\left[\begin{array}{cc} q_{z1} & 0\\ 0 & q_{z2} \end{array}\right]$$

(28b) $$R=\left[r\right]$$

The cost function Equation (29) is calculated by substituting Equations (21) and (22) to Equation (25).

(29) $$J\left(k\right)=\frac{1}{2}{\bar{u}}^{T}H\bar{u}+f^{T}\bar{u}=\;\frac{1}{2}{\bar{u}}^{T}\overset{H}{\overbrace{\left({\overset{=}{B}}^{T}{\overset{=}{Q}}_{x}\overset{=}{B}+{\overset{=}{B}}^{T}{\overset{=}{C}}^{T}{\overset{=}{Q}}_{z}\overset{=}{C}\overset{=}{B}+\overset{=}{R}\right)}}\bar{u}+\;\overset{f^{T}}{\overbrace{\left({\bar{x}}^{T}{\overset{=}{A}}^{T}{\overset{=}{Q}}_{x}\overset{=}{B}+{\bar{x}}^{T}{\overset{=}{A}}^{T}{\overset{=}{C}}^{T}{\overset{=}{Q}}_{z}\overset{=}{C}\overset{=}{B}+\hat{\bar{\Phi }}{\overset{=}{D}}^{T}{\overset{=}{Q}}_{z}\overset{=}{C}\overset{=}{B}\right)}}\bar{u}$$

The QP problem is defined in the following form:

(30a) $$\underset{u}{\min}\left[\frac{1}{2}{\bar{u}}^{T}H\bar{u}+f^{T}\bar{u}\right]$$

subject to

(30b) $$-0.5\;\left[rad\right]\leq u_{i}\;\leq 0.5\left[rad\right]\;\forall \;u_{i}\in \bar{u}$$

Finally, the solution of the QP problem is vector *u* which contains the optimal control input signals—in this case, the sequence of steering angles. Only the first element of vector *u* is applied to the vehicle, and the state measurement, the prediction of future states, and the solution of the quadratic programming problem are conducted in every time step. Although *u* is defined as roadwheel angle (RWA) in radians, the steering wheel angle (SWA) is shown in degrees in the figures below. The conversion from RWA to SWA is conducted based on the ratio of the steering system which is identified on the test vehicle. The constraint of 0.5 rad on the RWA expressed as SWA is 400 deg. Since the MPC is an online optimization process, the entire process needs to be able to run in real-time on the MicroAutoBox; the presented process can run for less than 1 ms as shown in Section 6.

## 5. Cascade MPC Formulation

The authors implemented a cascade MPC as suggested in [30], in which the steering system dynamics are modeled and controlled separately from the path-following MPC, by a second MPC. In this controller method, the first MPC—i.e., vehicle MPC—calculates the necessary angular velocity of the actual steering angle, and the second MPC—i.e., the steering MPC—is responsible for tracking it using a second-order steering system model for state prediction. In [30], the vehicle MPC uses system model Equation (31) for state prediction, where the state vector is *x = [*$e_{1}\;{\dot{e}}_{1}\;e_{2}\;{\dot{e}}_{2}$ $\delta_{ref}$*]^T^*, and the control input is $u={\dot{\delta }}_{ref}$.

(31) $$\dot{x}=Ax+Bu$$

$$A=\left[\begin{array}{ccccc} 0 & 1 & 0 & 0 & 0\\ 0 & -\frac{2C_{\alpha f}+2C_{\alpha r}}{mV_{x}} & \frac{2C_{\alpha f}+2C_{\alpha r}}{m} & \frac{-2C_{\alpha f}l_{f}+2C_{\alpha r}l_{r}}{mV_{x}} & \frac{2C_{\alpha f}}{m}\\ 0 & 0 & 0 & 1 & 0\\ 0 & -\frac{2C_{\alpha f}l_{f}-2C_{\alpha r}l_{r}}{I_{z}V_{x}} & \frac{2C_{\alpha f}l_{f}-2C_{\alpha r}l_{r}}{I_{z}} & -\frac{2C_{\alpha f}l_{f}^{2}-2C_{\alpha r}l_{r}^{2}}{I_{z}V_{x}} & \frac{2C_{\alpha f}l_{f}}{I_{z}}\\ 0 & 0 & 0 & 0 & 0 \end{array}\right]$$

$$B=\left[\begin{array}{c} \begin{array}{c} 0\\ 0 \end{array}\\ \begin{array}{c} 0\\ \begin{array}{c} 0\\ 1 \end{array} \end{array} \end{array}\right]$$

In the steering MPC, the second-order steering system is modeled by Equation (32), where the state vector is *x = [*$\delta_{act}\;{\dot{\delta }}_{act}$*]^T^*, and the control input is *u =* $\delta_{ref}$.

(32) $$\dot{x}=Ax+Bu$$

$$A=\left[\begin{array}{cc} 0 & 1\\ -a_{st0} & -a_{st1} \end{array}\right]$$

$$B=\left[\begin{array}{c} 0\\ b \end{array}\right]$$

The QP cost function is formulated and solved in both the vehicle MPC and in the steering MPC in the same way, as presented in Section 4. In the vehicle MPC, the applied *C* matrix is Equation (23b), the *Q_x_* matrix is Equation (27b), and the other matrices are identical. In the steering MPC, the reference is given by Equation (33) which is a vector that contains the reference angular velocity values calculated by the vehicle MPC.

(33) $$\Phi =\left[\begin{array}{cccc} {\dot{\delta }}_{ref1} & {\dot{\delta }}_{ref2} & \cdots & {\dot{\delta }}_{refNp} \end{array}\right]{\;}^{T}$$

In the steering MPC, the error vector needs to be modified (Equation (34)). The error vector is extended for the entire prediction horizon in the same way as in Equation (24). The applied ${\overset{=}{Q}}_{x}\in R^{2N_{p}\times 2N_{p}}$ and ${\overset{=}{Q}}_{z}\in R^{N_{p}\times N_{p}}$ are generated using the *Q_xst_* Equation (35) and *Q_zst_* Equation (36) matrices. The diagonal matrix $\overset{=}{R}$ is generated as in Equation (26c), using *r_st_*.

(34) $$z\left(k\right)=\left[{\dot{\delta }}_{ref}-{\dot{\delta }}_{act}\right]=Cx+D\phi =\left[\begin{array}{cc} 0 & -1 \end{array}\right]\left[\begin{array}{c} \delta_{act}\\ {\dot{\delta }}_{act} \end{array}\right]+\left[{\dot{\delta }}_{ref}\right]$$

(35) $$Q_{xst}=\left[\begin{array}{cc} 0 & 0\\ 0 & q_{xst} \end{array}\right]$$

(36) $$Q_{zst}=\left[q_{zst}\right]$$

The solution of the two QP problems is two vectors of length *N_p_* that contain the optimal reference angular velocity of the steering angle in the vehicle MPC, and the optimal reference steering angle in the steering MPC. The applied constraint on the vector of the optimal angular velocity of the steering angle is −2 rad/s ≤ ${\dot{\delta }}_{ref,i}$ ≤ 2 rad/s, $\forall \;{\dot{\delta }}_{ref,i}\in \;{\bar{\dot{\delta }}}_{ref}$ and the constraint on the optimal steering angle vector is −0.5 rad ≤ $\delta_{ref,i}$ ≤ 0.5 rad, $\forall \;\delta_{ref,i}\in {\bar{\delta }}_{ref}$.

The parameters of the test vehicle, the controllers, and the steering models are provided in Table 1.

## 6. Simulation and Experimental Results

**Figure 8:**
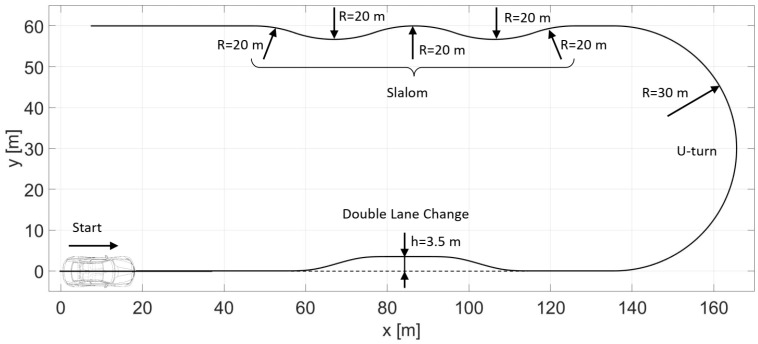
Reference path with the three main sections.

The simulation and the experimental results are conducted on the same reference path, on a flat asphalt surface. The path consists of three main sections, as shown in Figure 8, which are the double lane change, the u-turn, and the slalom segments.

The double lane change, which is a critical driving maneuver [36,37], has a 3.5 m lateral evasion where the entering and leaving sections are generated by using clothoid transition curves. The u-turn is a semicircle with a 30 m radius. The slalom segment is constructed by five arcs with a 20 m radius. At the double lane change section, the curvature of the path is increasing continuously. However, at the u-turn and the slalom sections, no transitional arcs are applied; thus, there are discontinuities in the curvature which makes it harder for the vehicle to follow the path.

Furthermore, the reference path contains two straight line segments at the beginning and at the end of the path. The straight section at the beginning is intended to provide the necessary distance for the vehicle to accelerate to the required speed. The reference velocity of the vehicle is reached in the straight section and kept on this value during the entire path by a PI controller. The straight section at the end is applied to ensure the vehicle stabilizes after the slalom section. The throttle is only released at the final straight section.

The simulation and the experimental tests are conducted using both the vehicle model without steering dynamics and the enhanced vehicle model including the steering dynamics for state prediction. During the tests, the target velocity of the vehicle is increased until the controller is unable to follow the path. The target velocity is started at 30 km/h and increased in 10 km/h steps until the applicability limit of the controller is reached and the controller becomes unable to follow the path. The tuning parameters of the controller are constant during the tests. Furthermore, the same parameters are used in the simulation and during the experimental tests. The prediction horizon is unchanged as well and is identical in every setup; thus, the same identically tuned and parametrized controllers are tested in the simulation and experimental environments.

### 6.1. Simulation and Experimental Results Excluding Steering Dynamics

In this section, the presented simulation and experimental results are both conducted using the vehicle model which excludes the steering dynamics for state prediction. The results are summarized in Table 2, where *e_avg_* and *ϕ_avg_* are the average lateral and orientation errors, respectively, and *e_max_* and *ϕ_max_* are the maximal lateral and orientation errors, respectively.

**Figure 9:**
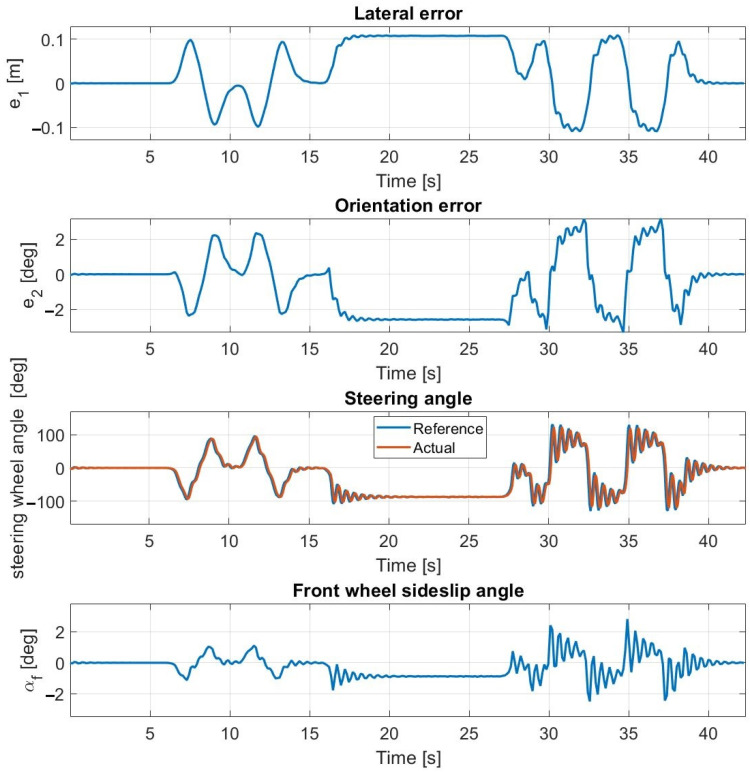
Simulation results at 30 km/h—without steering dynamics.

**Figure 10:**
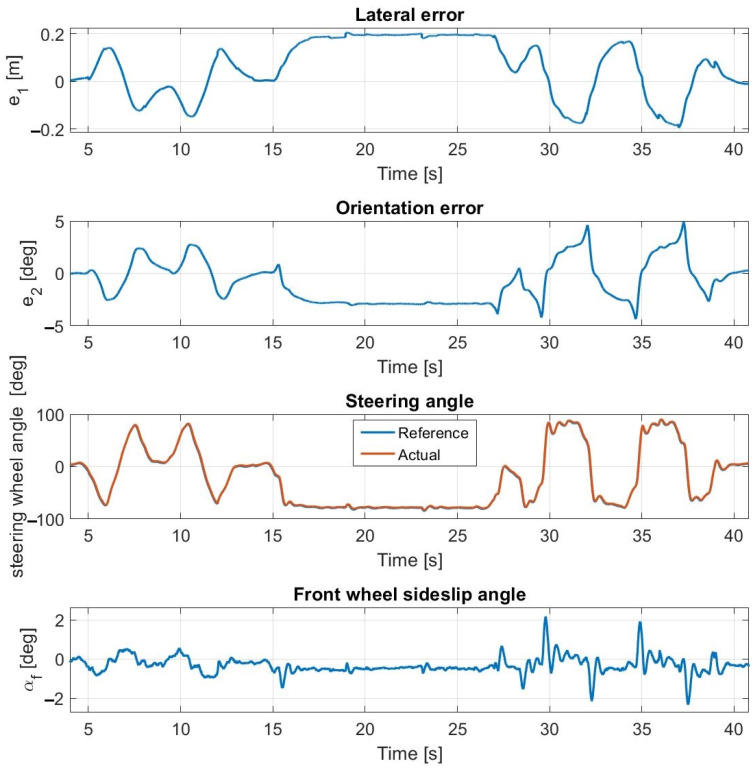
Measurement results at 30 km/h—without steering dynamics.

The results of the first test case conducted at 30 km/h are shown in Figure 9 and Figure 10. The simulation and the test results are highly close to each other, the average error values are low, and the maximal values are appropriate as well. For both the simulation and the measurements, the sideslip angle falls within the same interval (≤3.5 degrees) which is the linear section of the tire characteristic. An oscillation phenomenon appears in the steering angle which is much more significant during simulation rather than experimental tests. The presence of the oscillation is assumed to be the result of the neglect of the steering dynamics.

At a speed of 40 km/h, the controller still operates stably and the lateral and orientation errors are still low. The amplitude of the oscillation is increased compared to the tests conducted at 30 km/h which made the travel inconvenient for the passengers. In the slalom section, the sideslip angles reach the nonlinear region of the tire characteristics (≥3.5 degrees) which further increases the oscillation.

**Figure 11:**
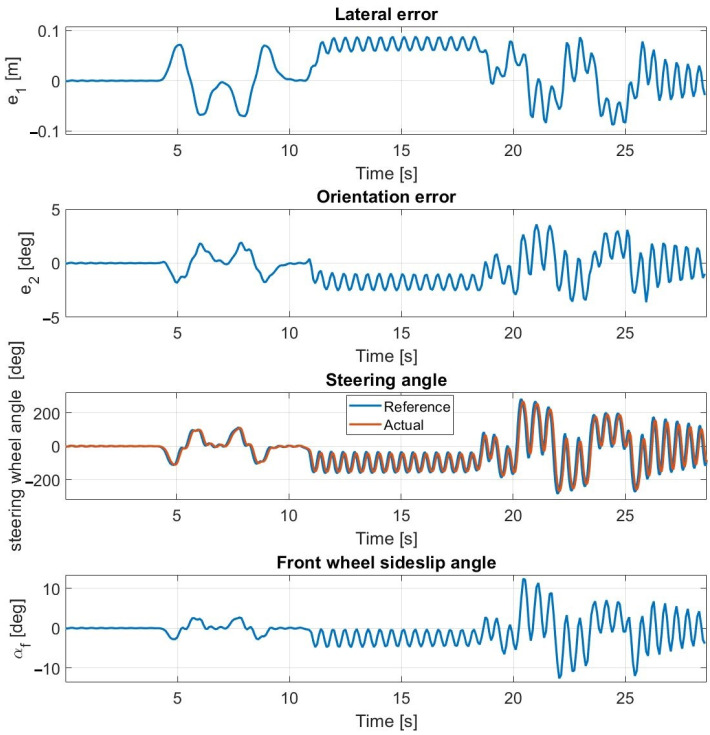
Simulation results at 45 km/h—without steering dynamics.

**Figure 12:**
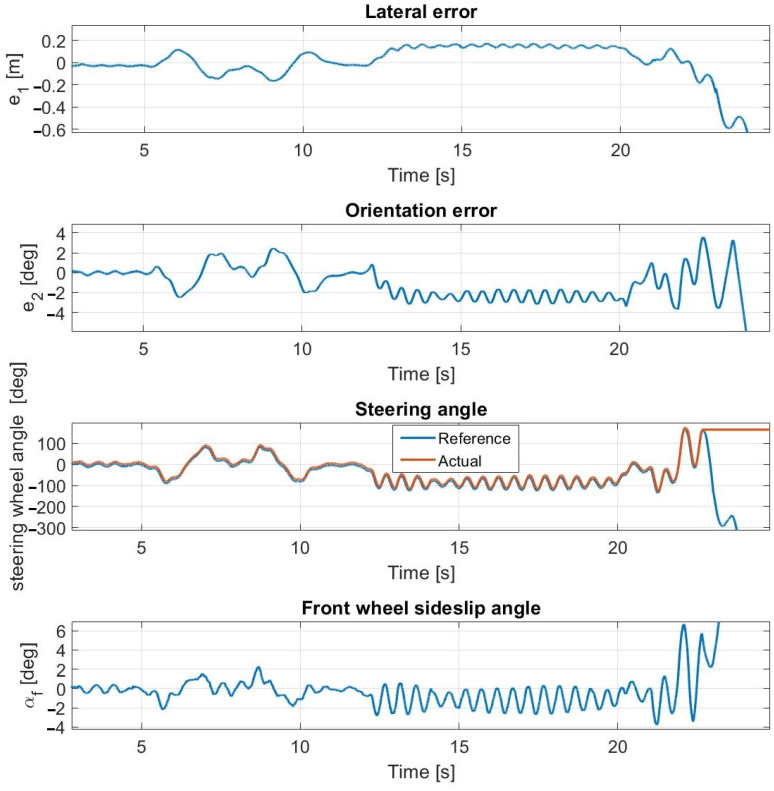
Measurement results at 45 km/h—without steering dynamics.

The highest speed at which the tests can be performed is 45 km/h as shown in Figure 11 and Figure 12. In the simulation, the controller can drive the vehicle along the entire path; however, during the experimental tests, the amplitude of the oscillation becomes so high that the test is stopped—despite the fact that the controller can follow the path and the error values are low, the results are not applicable. The appearance of the oscillation in the simulation correctly predicts that it will also appear in the experimental tests.

As seen in Table 2, the simulation results of the lateral and orientation errors are an accurate approximation of the measurement results, however, the average and maximal errors are slightly greater.

The appearing oscillation is primarily due to the neglect of the actuator dynamics which are the dynamics of the steering system in this case, and secondly, at higher speeds, due to the prediction inaccuracy of the linear tire model. Tuning the parameters of the controller to be less sensitive for the errors results in a decrease in the oscillation; however, the accuracy of the path following significantly deteriorates as well.

### 6.2. Simulation and Experimental Results including Steering Dynamics

In this section, the simulation and experimental results of the application of the steering models in the MPC for state prediction are presented, as shown in Table 3.

**Figure 13:**
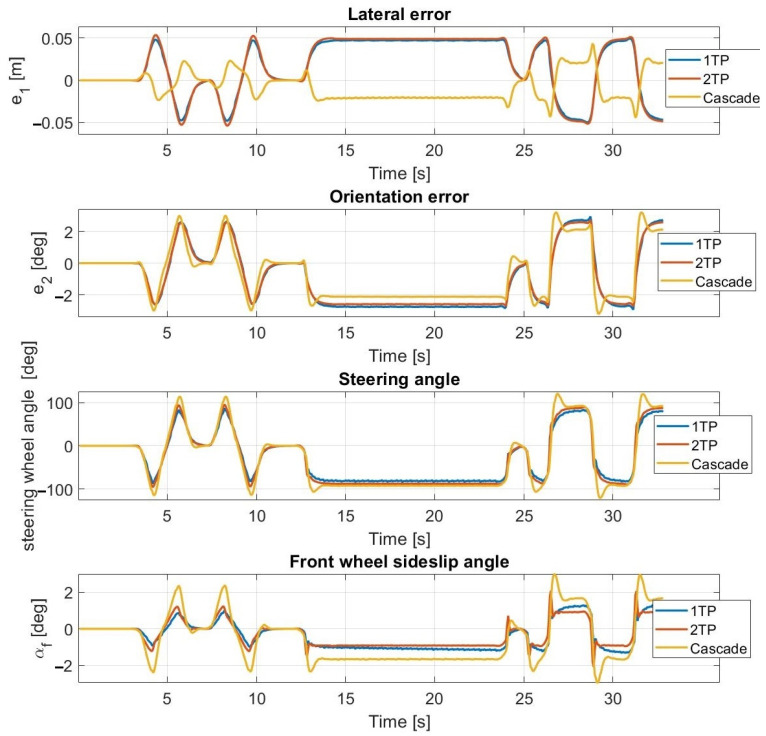
Simulation results at 30 km/h—including steering dynamics.

**Figure 14:**
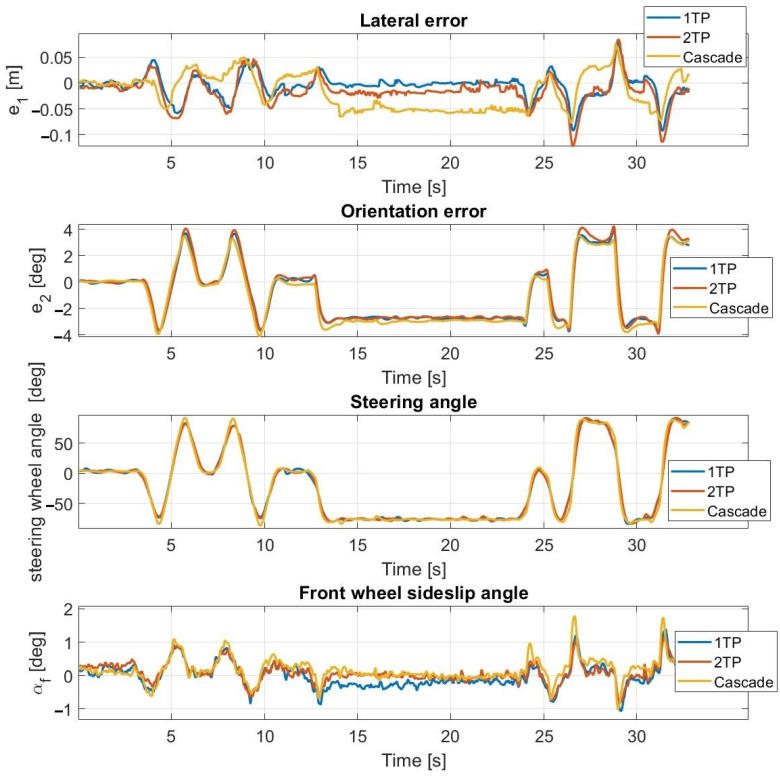
Measurement results at 30 km/h—including steering dynamics.

At 30 km/h (Figure 13 and Figure 14) and at 40 km/h, the simulation results approximate the measurement results accurately; however, the average lateral error is greater during the measurements than in the simulation. The oscillation is entirely missing as a result of the inclusion of the steering dynamics.

**Figure 15:**
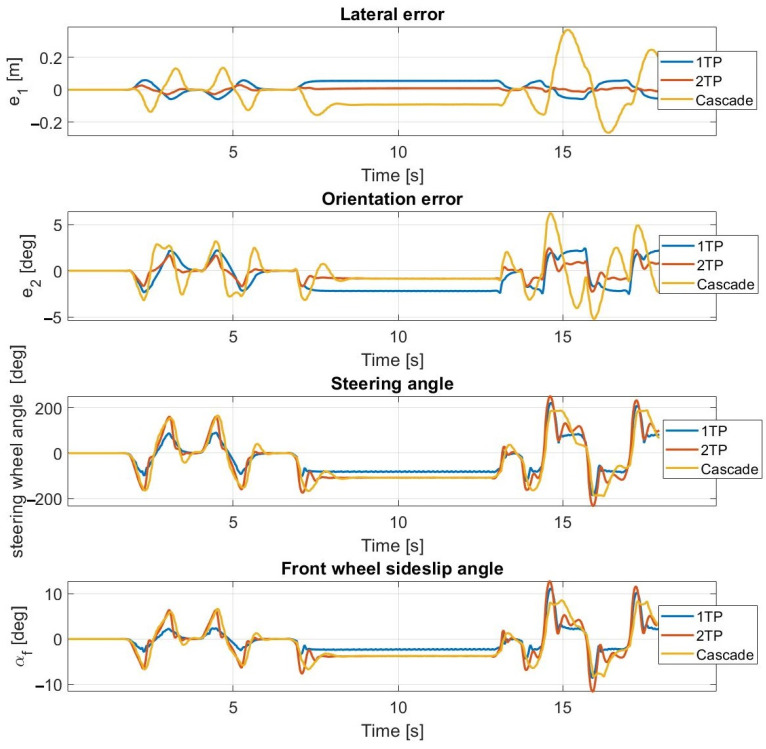
Simulation results at 55 km/h—including steering dynamics.

**Figure 16:**
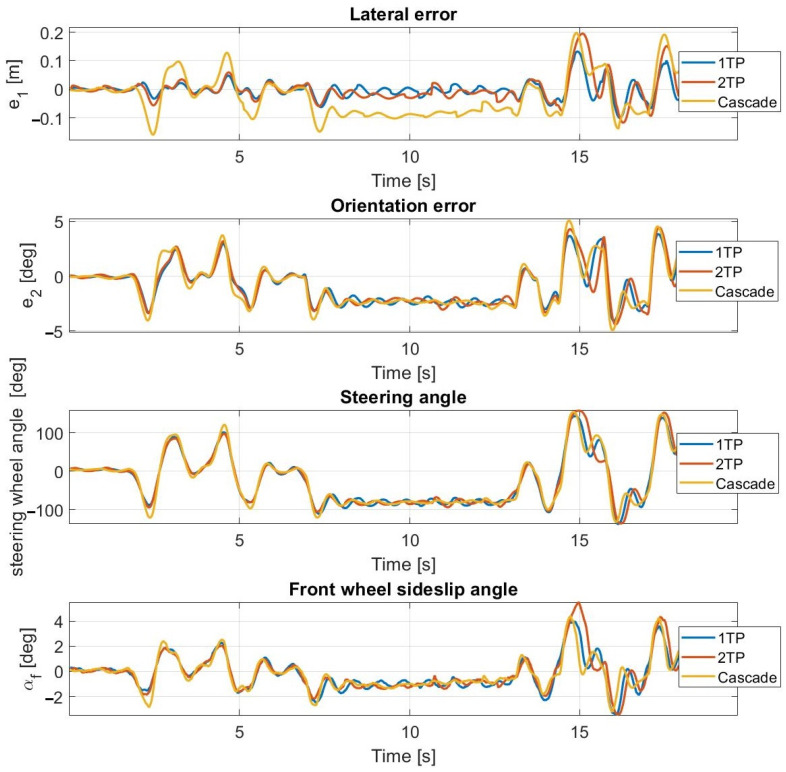
Measurement results at 55 km/h—including steering dynamics.

At 50 km/h and at 55 km/h (Figure 15 and Figure 16), the controller still operates stably and accurately, proven by the simulation results being close to the measurement results. The consideration of the steering dynamics enables the controller to drive the vehicle along the path at enhanced speeds, while the oscillation appears at first only at 55 km/h. At this speed, the sideslip angles reach and exceed the 3.5 degrees limit, which is the applicability limit of the applied linear tire models. Hence, the appearing oscillation is the result of invalid state prediction caused by exceeding the limit of the applied linear tire model in the prediction model. The application of the second-order steering model does not result in a performance improvement, which is due to the high torque of the steering robot. At 50 km/h, the cascade MPC provides acceptable performance, but at 55 km/h, high errors appear.

**Figure 17:**
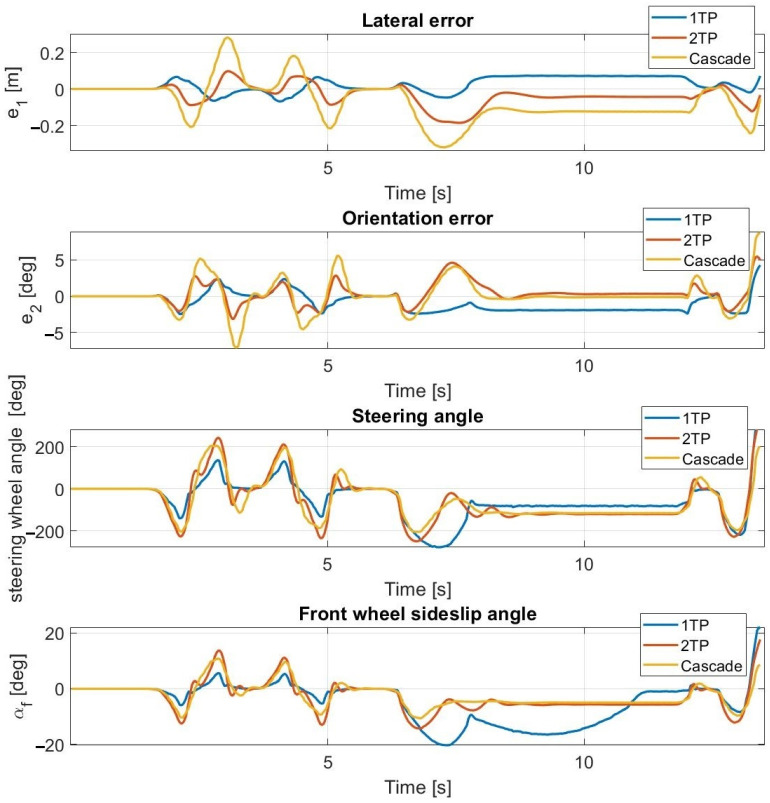
Simulation results at 60 km/h—including steering dynamics.

**Figure 18:**
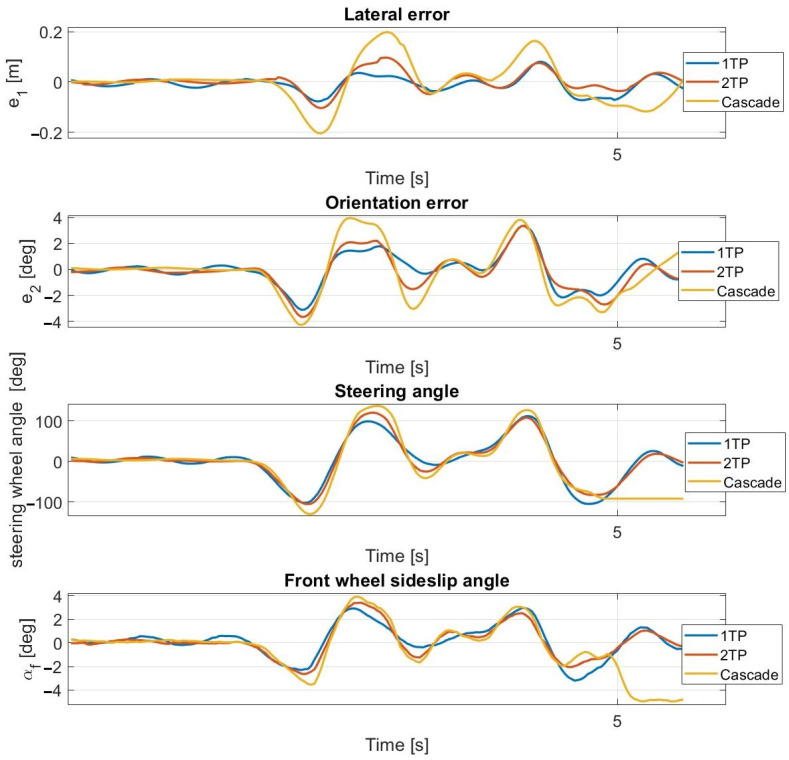
Measurement results at 60 km/h—including steering dynamics.

At 60 km/h (Figure 17 and Figure 18), the sideslip angles of the tires reach the limit of applicability; hence, all of the applied controllers lose control over the vehicle. The vehicle can perform the double lane change and the u-turn sections, using the first-order and the second-order steering models; however, the controller loses control in the slalom section, in both simulation and experimental tests. During the experimental tests, the measurements are plotted until the u-turn section, because the cascade MPC loses control here.

Please note that at 60 km/h, at the ending section of the tests, the lateral and orientation errors are high. Furthermore, the simulation and the experimental test are not aborted at exactly the same path point.

These have a considerable effect on the calculated average errors; hence, the simulation and the test results at 60 km/h do not show as high a degree of coincidence as at other speeds.

The controllers lose control in the slalom section due to the change in the curvature being high there, which results in a large steering angle along with a high sideslip angle. Moreover, the vehicle reaches its driving limit due to the high curvatures of the slalom section—even with a human driver. Furthermore, until the slalom section, the controllers using the first-order and the second-order steering models follow the path highly accurately despite the instability. The poor performance of the cascade MPC is the result of the exclusion of the steering dynamics from the vehicle MPC’s plant model.

Regarding the simulation results, overall, they can accurately represent the expected behavior of the vehicle. In the 1TP test cases, the average lateral errors are smaller during the tests than in the simulations, but apart from that, the errors were slightly greater in the experimental tests than in the simulations; however, the differences remain small. Applying the same controller parameters and using the vehicle model, which is parametrized based on measurements, the simulations give accurate results, which proves that the applied vehicle model is correct and is suitable for in-simulation controller development.

Based on the simulation and the experimental results, the authors experienced that the dynamic model of the vehicle and the steering system should be integrated into the same MPC, for better performance.

**Figure 19:**
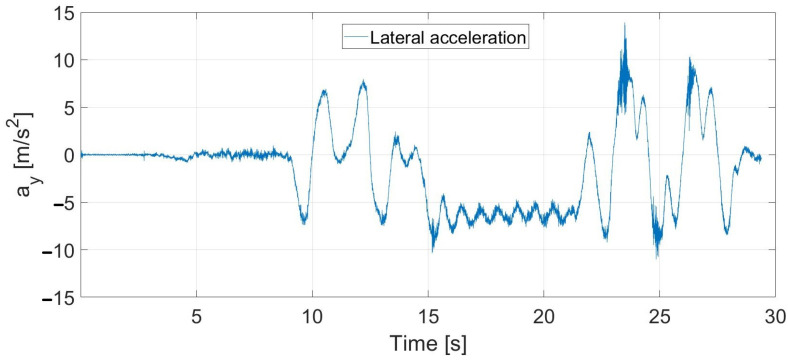
Lateral acceleration at 55 km/h, 1TP steering model.

The lateral acceleration during the experimental test conducted at 55 km/h using the 1TP steering model is shown in Figure 19. The maximal lateral acceleration is about 9 m/s^2^ at the slalom section; here, the signal noise is generated by the high sideslip of the tires. In the double lane change section, the lateral acceleration reaches about 7.5 m/s^2^, where the controller can still drive the vehicle stably. The unstable behavior starts when the sideslip reaches 3.5 degrees, as shown in Figure 16.

**Figure 20:**
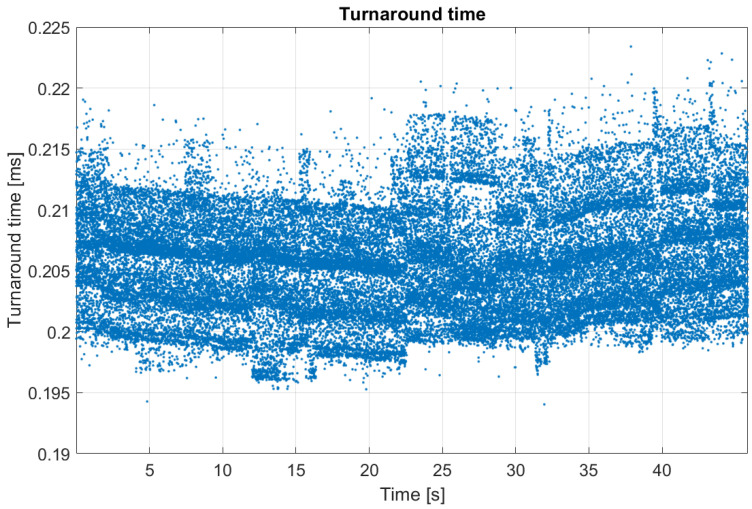
The time requirement of the control software on the MicroAutoBox II during the “no steering dynamics” test case.

The computational requirements are examined solely in those cases when the controller is run on the MicroAutoBox II rapid prototyping unit. The values of the turnaround time provided by the MicroAutoBox II are shown in Table 4, while Figure 20 shows the turnaround time values for a measurement in which no steering dynamics are considered. The turnaround time values show the time which is needed to run the entire control model—including the state measurement, the prediction of the future states, and the solution of the QP problem. By increasing the dimension of the *x* state vector of the plant model, the computational requirement is increased but remains under 1 ms which is sufficient for running the controller with a 1 ms time step, which is the desired target value in the field of controller development. The results show that the proposed LPV-MPC controller has a low computational requirement compared to other methods, e.g., a NLMPC described in [14] where the computational time is 5–35 ms, or LTV-MPC solutions, e.g., in [15], where 100–600 ms is needed for running the controller, depending on the applied vehicle model, or in the other LTV-MPC [18], where the execution time is about 2 ms.

## 7. Conclusions and Future Work

In this paper, an MPC approach for automated vehicle path following is presented. A vehicle model for state prediction and a novel method for reference state generation is proposed where the reference states are defined by the desired lateral error and the orientation error regarding the current state of the vehicle.

Regarding the applied vehicle model, when the steering dynamics are neglected at the state prediction, the lateral error is increased at each test case and the controller starts to oscillate at around 40 km/h, and for higher speeds, it is unable to follow the path. When the steering dynamics are considered for the state prediction, the limit of the controller is increased to up to 55 km/h—for this test vehicle. Consequently, with the inclusion of the model of the steering system in the prediction model, the performance of the controller is significantly increased.

In this article, four different models are applied for state prediction; the best performance is achieved when the first-order steering model is coupled with the vehicle model in a single MPC. There are no significant differences when applying the first-order and the second-order steering models; the application of the first-order system provided a slightly more accurate path-following. However, the modeled steering system has very fast dynamics, and a small time constant, which is due to the high torque of the steering robot. A cascade MPC structure is also examined, where the steering dynamics is considered separately from the vehicle dynamics in a second MPC. This solution provided better performance than when the steering dynamics were neglected and solely the vehicle dynamics were considered for state prediction. However, according to our experiments, the best performance can be reached when the dynamics of the vehicle and the steering system are integrated into one model, applying one MPC. The performance degradation of the cascade MPC is due to the incapability of the vehicle MPC for taking into account the steering dynamics during the state prediction. Based on the conducted tests, the authors suggest integrating the dynamics of the vehicle and the steering system into one MPC for the best path-following performance; however, this results in a slightly higher computational demand.

As future research opportunities, from a modeling point of view, the application of a more detailed steering system model would be able to further increase the performance of the controller, especially if the steering actuator does not have as high torque as the applied one. The controller uses a linear vehicle and tire model for state prediction, which leads to inaccurate state prediction when the vehicle operates at higher lateral accelerations. To overcome this issue the accuracy of the state prediction needs to be enhanced, which could be reached by applying a successive linearization method in an LTV-MPC structure, or by using NLMPC. The application of the nonlinear vehicle and tire models in a LTV-MPC or NLMPC structure coupled with a more detailed model of the steering system would be able to increase the application limit of the controller. Furthermore, a LTV-MPC is also under development by the authors to be able to handle the vehicle when the tires operate in the nonlinear region. The consideration of the backlash of the steering system by the path-following controller is also a potential research field. As the vehicle is driven in a more dynamic maneuver, e.g., during higher lateral accelerations or at higher speeds, the effect of backlash becomes more important, which needs to be considered in the future. Moreover, a potential research opportunity is the consideration of the actuator fault as discussed in [6], and making the controller more adaptive, e.g., changing the value of the prediction horizon, the control horizon, and the controller gains depending on the velocity of the vehicle as presented in [8]. The online estimation of the tire cornering stiffness [8], the friction coefficient [8] and other vehicle parameters can also improve the performance of the controller.

## Figures and Tables

**Table 1:** Parameter values of the vehicle, the controller, and the steering models.

Name	Value	Sign and Dimension
Vehicle parameters		
mass of the vehicle	1810	m (kg)
vehicle moment of inertia around the *z*-axis	2500	I_z_ (kgm^2^)
cornering stiffness of the front tires	150,000	C_αf_ (N/rad)
cornering stiffness of the rear tires	250,000	C_αr_ (N/rad)
friction coefficient (lateral)	1	μ (-)
distance between the center of gravity and the front axle	1.35	a (m)
distance between the center of gravity and the rear axle	1.37	b (m)
Controller parameters		
prediction horizon	10	N_p_ (pcs)
control horizon	10	N_c_ (pcs)
weight of lateral error	0.85	q_x1_ (-)
weight of orientation error	1.1	q_x2_ (-)
weight of the deviation from the lateral reference	0.85	q_z1_ (-)
weight of the deviation from the orientation reference	1.1	q_z2_ (-)
weight of steering MPC error	0.5	q_xst_ (-)
weight of the deviation from the steering angular velocity reference	0.5	q_zst_ (-)
weight of the control input	0.7	r (-)
weight of control input in the steering MPC	0.8	r_st_ (-)
Steering model parameters		
first-order model: Tst	0.012	(s)
second-order model: a_st1_	248.06	(-)
second-order model: a_st0_	21,915.56	(-)
second-order model: b	21,851.67	(-)

**Table 2:** Lateral and orientation error results—excluding steering dynamics.

	Speed (km/h)	e_avg_ (m)	e_max_ (m)	ϕ_avg_ (deg)	ϕ_max_ (deg)
Simulation	30	0.078	0.134	1.437	4.183
	40	0.083	0.160	1.299	4.110
	45	0.161	0.548	1.382	13.486
Measurement	30	0.10	0.20	1.66	4.97
	40	0.11	0.23	1.46	5.36
	45	0.15	2.84	1.81	22.74

**Table 3:** Lateral and orientation error results—including first-order, second-order steering dynamics and cascade MPC.

Speed (km/h)		e_avg_ (m)	e_max_ (m)	ϕ_avg_ (deg)	ϕ_max_ (deg)
30 km/h	Simulation—1TP	0.026	0.054	1.559	2.869
	Measurement—1TP	0.016	0.099	1.923	4.203
	Simulation—2TP	0.027	0.055	1.426	2.817
	Measurement—2TP	0.025	0.123	2.103	4.343
	Simulation—cascade	0.013	0.044	1.317	3.212
	Measurement—cascade	0.031	0.079	2.137	4.147
40 km/h	Simulation—1TP	0.025	0.052	1.454	2.664
	Measurement—1TP	0.013	0.088	1.783	4.194
	Simulation—2TP	0.022	0.045	1.190	2.426
	Measurement—2TP	0.015	0.097	1.931	4.121
	Simulation—cascade	0.022	0.059	1.363	3.567
	Measurement—cascade	0.041	0.091	1.945	4.322
50 km/h	Simulation—1TP	0.024	0.050	1.308	2.524
	Measurement—1TP	0.011	0.075	1.673	3.764
	Simulation—2TP	0.013	0.033	0.828	1.911
	Measurement—2TP	0.016	0.091	1.771	3.880
	Simulation—cascade	0.040	0.126	1.094	4.620
	Measurement—cascade	0.057	0.149	1.877	4.811
55 km/h	Simulation—1TP	0.028	0.058	1.207	2.506
	Measurement—1TP	0.021	0.132	1.587	4.096
	Simulation—2TP	0.006	0.030	0.569	2.446
	Measurement—2TP	0.029	0.195	1.666	4.466
	Simulation—cascade	0.053	0.303	1.224	6.534
	Measurement—cascade	0.064	0.218	1.793	5.223
60 km/h	Simulation—1TP	0.036	0.332	1.164	4.984
	Measurement—1TP	0.041	0.577	1.501	7.844
	Simulation—2TP	0.042	0.195	0.890	5.474
	Measurement—2TP	0.037	0.338	1.582	6.761
	Simulation—cascade	0.092	0.448	1.286	8.704
	Measurement—cascade	0.059	0.204	1.386	4.288

**Table 4:** Turnaround time of the control model.

	Minimal (ms)	Average (ms)	Maximal (ms)
No steering dynamics	0.1940	0.2059	0.2250
1TP steering model	0.2919	0.3091	0.3328
2TP steering model	0.2966	0.3095	0.3339
Cascade MPC	0.2918	0.3058	0.3229

## Data Availability

Not applicable.
